# Muscle in the caterpillar *Manduca sexta* responds to an immune challenge, but at a cost, suggesting a physiological trade-off

**DOI:** 10.1242/jeb.245861

**Published:** 2023-07-25

**Authors:** Shelley A. Adamo, Emily Corkum, Jongseok Kim, Tingyat M. Lee, Dylan W. Miller, Sungwoo Song, Christopher Wright, Isaac D. Zacher, Jeffrey S. Zbarsky, Laura E. McMillan

**Affiliations:** Department of Psychology and Neuroscience, Dalhousie University, Halifax, NS, Canada, B3H 4R2

**Keywords:** Ecoimmunology, Predator stress, Pathogen, Trade-offs, Lepidoptera

## Abstract

Although skeletal muscle is a specialized tissue that provides the motor for movement, it also participates in other functions, including the immune response. However, little is known about the effects of this multitasking on muscle. We show that muscle loses some of its capacity while it is participating in the immune response. Caterpillars (*Manduca sexta*) were exposed to an immune challenge, predator stress or a combination of immune challenge and predator stress. The expression of immune genes (*toll-1*, *domeless*, *cactus*, *tube* and *attacin*) increased in body wall muscle after exposure to an immune challenge. Muscle also showed a reduction in the amount of the energy storage molecule glycogen. During an immune challenge, the force of the defensive strike, an important anti-predator behaviour in *M. sexta*, was reduced. Caterpillars were also less able to defend themselves against a common enemy, the wasp *Cotesia congregata*, suggesting that the effect on muscle is biologically significant. Our results support the concept of an integrated defence system in which life-threatening events activate organism-wide responses. We suggest that increased mortality from predation is a non-immunological cost of infection in *M. sexta*. Our study also suggests that one reason non-immunological costs of infection exist is because of the participation of diverse organs, such as muscle, in immunity.

## Teaser

**Highlighted Article:** Muscle contributes to the immune response; however, this participation reduces muscle function and increases mortality from predation. Sharing resources optimizes function, but comes at a cost.

## INTRODUCTION

Organisms are composed of physiological systems specialized to perform specific tasks (e.g. muscles allow animals to move). However, physiological systems are deeply interconnected, allowing them to play key roles in seemingly unrelated functions (Adamo, 2022). Muscle, for example, contributes to immune function in both insects (Yang and Hultmark, 2016, 2017) and mammals (Rogeri et al., 2020), in part by providing resources to the immune system (Yang and Hultmark, 2017). Being able to recruit additional resources during life-threatening challenges can allow animals to maximize their response (Adamo, 2022), but how shifts in resources between muscle and immunity impact muscle is unclear. We explored how muscle responds to an immune challenge in the caterpillar *Manduca sexta*, focusing on the potential costs of this multitasking.

Insect immune systems rely on both cellular and humoral mechanisms (Buchon et al., 2014). Two major components are: (i) the haemocytes, i.e. circulating blood cells that engulf and sequester pathogens (Eleftherianos et al., 2021a), and (ii) the fat body, i.e. an organ that produces important immune defence molecules such as antimicrobial peptides (AMPs; Skowronek et al., 2021). Immune responses are regulated by insect cytokines (Vanha-aho et al., 2016), and other immune modulators, such as molecules that recognize pathogen-associated molecular patterns (PAMPS) and danger-associated molecular patterns (DAMPS) (Krautz et al., 2014). These molecules signal pathogen presence and/or damage (Krautz et al., 2014). The combined cellular and humoral immune defences efficiently destroy most pathogens, allowing insects to flourish even in environments replete with potentially pathogenic organisms. However, mounting an immune defence is resource intensive (Ardia et al., 2012). The resource costs are best known for *Drosophila melanogaster*. Activating an immune response induces a metabolic shift in haemocytes, resulting in an increased need for glucose (Krejčová et al., 2019). During an encapsulation response, haemocytes consume 27% of the overall glucose budget in *D. melanogaster* (Bajgar et al., 2015). These changes in metabolism allow for a rapid and effective response (Bajgar and Dolezal, 2018). A rapid response is critical because resolving an infection quickly is important for overcoming a pathogen (Duneau et al., 2017). The extra glucose is provided, in part, by the breakdown of glycogen in the fat body (Dolezal et al., 2019), which is also an energy storage organ (Skowronek et al., 2021). There are few other ready sources of glucose in the insect (Mattila and Hietakangas, 2017). One of those sources is muscle (Mattila and Hietakangas, 2017).

In larval *Drosophila*, muscle glycogen helps fuel the immune response (Yang and Hultmark, 2017). Connections between muscle and immune tissues allow muscle to play a coordinated role in immune defence (Green et al., 2018; Eleftherianos et al., 2021a). Muscle releases signalling molecules (e.g. Wingless and myokinins) that help regulate energy storage in the fat body (Bretscher and O'Connor, 2020). Muscle also has indirect effects on immune function via the suppressive effects of myokinins on insulin-like peptide (ILP) production in the brain (Alfa and Kim, 2016). ILPs play a role in the fat body's immune response (Musselman et al., 2018). Immune tissues also communicate with muscle. In larval *Drosophila*, immune tissues release cytokines such as Unpaired (Upd) during an immune response (Yang and Hultmark, 2017). Upd binds with the Domeless receptor on muscle, activating the Jak/Stat pathway (Yang and Hultmark, 2017). This activation reduces glycogen stores in muscle (Yang and Hultmark, 2017). It also decreases lipid uptake into muscle, increasing energy availability for the immune system (Dolezal et al., 2019; Bajgar et al., 2021). These changes in metabolism are crucial for an effective immune response (Bajgar et al., 2021). In addition to providing resources, bacterial infection induces muscle to produce AMPs in adult *Drosophila* (Chatterjee et al., 2016). Muscle also expresses genes for pathogen recognition molecules (e.g. in *Antheraea pernyi*, Lepidoptera; Liu et al., 2016). Therefore, muscle does more than donate energy substrates, it also appears to assist the immune system in the production of some immune molecules.

Muscle's contribution to immune function appears to be necessary for a robust defence (Zhao and Karpac, 2021). However, insects live in a world filled with predators, and effective muscle function is critical for their anti-predator behaviour (e.g. Robertson et al., 2013). If muscle participates in immunity, how does this participation impact its ability to produce anti-predator behaviour? There is indirect evidence in insects that muscular ability declines during an immune response. For example, activating immune responses reduces anti-predator behaviour, leading to increased predation in crickets (Otti et al., 2012). Although part of the decline in anti-predator behaviour appears to be due to oxidative damage to muscles generated by the immune system (e.g. damselflies; Janssens and Stoks, 2014a,b), some of the reduction may also be due to shifting resources from muscle to immunity.

To determine whether there are potential costs to muscle's participation in immune defence, we first investigated whether *M. sexta* muscle responds to pathogen exposure. We tested whether exposure to pathogens induced an upregulation of expression of canonical immune genes in the toll pathway, such as *toll-1*, *tube*, *cactus* and the AMP *attacin* (Buchon et al., 2014; Cao et al., 2015). We also tested for upregulation of *domeless*, the gene for the receptor for the Jak/Stat pathway.

We also examined the effect of an immune challenge on the expression of *glycogen synthase* and *glycogen phosphorylase*, genes involved in the synthesis and breakdown of glycogen, a major storage molecule in muscle (Bretscher and O'Connor, 2020). To test whether a combined predator and pathogen challenge might be more damaging to muscle than a single challenge, we also examined the expression of *glutathione-S-transferase* 1 (*GST-1*), a gene for an enzyme that is able to both detoxify and protect against oxidative stress (Kim et al., 2011). The larvae of the damselfly *Coenagrion puella* suffer greater oxidative stress-generated damage when exposed to a combined pathogen and predator challenge (Janssens and Stoks, 2014a).

To explore possible cross-organ connections, we examined the expression of the genes for both *toll-1* (a receptor that activates the toll pathway in *M. sexta*; An et al., 2010) and *InR* (a receptor that activates the insulin-signalling pathway; Chowański et al., 2021) in muscle, fat body and the central nervous system (CNS). In the CNS, we examined gene expression in abdominal ganglia 3, 4 and 5. These are the portions of the CNS that are closest to the excised muscle and fat body used in this study. We also examined expression in the supraoesophageal ganglion, the part of the CNS located in the head capsule, which is involved in advanced information processing (Nation, 2008).

We then explored possible costs of muscle's immune participation by assessing whether the glycogen content of body wall muscle was reduced during an immune challenge. We also tested whether an immune challenge impacts the caterpillar's defensive strike, its primary anti-predator behaviour (Walters et al., 2001). The defensive strike requires strong, coordinated responses from body wall muscles (Mukherjee et al., 2020). We tested both the strength of this behaviour and a caterpillar's ability to evade an ecologically relevant predator, the wasp *Cotesia congregata* (Kester and Barbosa, 1991), while mounting an immune response.

Pathogens and predators are both life threatening, and each activates an expensive physiological response that requires multiple organ systems (i.e. immune and fight-or-flight responses) (Adamo, 2020). Facing these two challenges simultaneously is probably common in the natural environment, as predators do not stop seeking prey because the prey are ill. For *M. sexta*, predator attack is a common occurrence (Bernays, 1997), and pathogens are ubiquitous. The response of the insect immune system to a combined predator/pathogen challenge suggests that physiological networks have different configurations depending on whether an organism is exposed to single or multiple threats (Adamo, 2020, 2022). For example, the stress neurohormone octopamine depresses lysozyme-like activity in the haemolymph of the cricket *Gryllus texensis* (Adamo, 2010). However, when pathogens are present, octopamine enhances lysozyme-like activity (Adamo, 2010). We predicted that muscle's response to an immune challenge would also be context dependent. We predicted that muscle would show less ability to support the immune system when the risk of predation was high. To test this prediction, we examined whether exposure to a mock predator attack affects the response of muscle to an immune challenge.

## MATERIALS AND METHODS

### Animals

Our *Manduca sexta* (Linnaeus 1763) colony was derived from eggs from Great Lakes Hornworm (Romeo, MI, USA). Periodically, new eggs from Great Lakes Hornworm were added to the colony. Animals were maintained as described in Adamo et al. (2016). Caterpillars were fed an artificial diet designed for *M. sexta* by Frontier Agricultural Sciences (Newark, DE, USA; F9783B) for the first four instars, except for a small number of caterpillars in the glycogen muscle study (see Supplementary Materials and Methods) that were fed on a *M. sexta* diet sourced from Great Lakes Hornworm. Caterpillars were reared in individual cups (96 ml, 6 cm diameter×3.6 cm height) after the first instar until the fourth instar. Upon moulting to the fifth instar, caterpillars were placed in larger cups (473 ml, 8 cm diameter×11 cm height) that contained stiff black mesh to raise them above the frass. Once caterpillars entered the fifth instar, they were given food diluted 1:3 with non-nutritive cellulose to provide nutrition closer to what they would receive in the field (e.g. lipid; Fernando-Warnakulasuriya et al., 1988). Trade-offs can be masked by unnaturally rich diets (Adamo et al., 2016) and we wanted to increase our ability to see any potential trade-offs between muscle participation in immunity and anti-predator behaviour. We switched the diet when caterpillars entered the fifth instar because this is the period in which weight gain is most rapid, and for which baseline data on the physiological effects are available (Reynolds et al., 1986; Timmins et al., 1988; Adamo et al., 2016). The reduced diet allows for normal caterpillar development, and caterpillars maintain growth by increasing food consumption of the lower nutrition diet (Timmins et al., 1988), similar to their behaviour when provided with fresh tobacco leaves (Reynolds et al., 1986). Caterpillars on the diluted diet have less lipid, glucose, protein and trehalose in their haemolymph compared with caterpillars maintained on the full nutrition diet, demonstrating a significant reduction in available resources (Adamo et al., 2016). Caterpillars were kept between 21 and 23°C, on a 12 h:12 h light:dark cycle, with a relative humidity of 45–70%.

Wasps (*C. congregata*) were shipped to Dalhousie University as cocoons from the lab of Dr Karen Kester, Virginia Commonwealth University, USA, and were maintained as previously described (Bredlau and Kester, 2015).

The study was approved by the University Committee on Laboratory Animals (I-18-06c) and was in accordance with the guidelines of the Canadian Council on Animal Care.

### Description of the five treatment groups

Upon reaching the fifth instar (i.e. day 0), caterpillars were weighed and randomly assigned to one of five groups: immune challenge, predator challenge, combined immune and predator challenge, sham-injected or control. Treatments were adapted from Adamo et al. (2017). Briefly, on the first day of the fifth instar, immune-challenged caterpillars were injected with 40 µl of three heat-killed pathogens [equivalent to the LD50 dose of live pathogen: *Beauveria bassiana* (fungus), *Bacillus cereus* (Gram-positive bacteria), *Serratia marcescens* (Gram-negative bacteria); see Adamo et al., 2016 for details]. This volume (40 µl) represents less than 2% of the blood volume of a typical fifth instar day 2 caterpillar (Adamo et al., 2007) and has no effect on weight gain in *M. sexta* (Adamo, 2005). Sham-injected caterpillars were pierced with a sterile needle. The predator-challenged group had their A6 proleg squeezed 8 times within 30 s with flexible forceps or were poked with a 4.72–4.93 N von Frey filament to induce a defensive strike. This treatment was repeated 4 times within 2 h. Animals that did not respond with a defensive strike (see description below) were excluded from the study. The combined challenge group were given both an immune challenge and predator challenge during the same period. The control group was unmanipulated. These treatments were repeated for each group on the morning of the second day of the fifth instar. Three hours after the second treatment, the caterpillars were weighed again and were then used in one of the following experiments. Therefore, both the predator and pathogen challenge were given over 2 days. For a short-lived animal like *M. sexta*, 2 days represents approximately 8–9% of their larval life (Reinecke et al., 1980), depending on the effect of each stressor on development time. For example, chronic predator stress increases *M. sexta* development time by about 1 day (Adamo et al., 2017). Therefore, the application of these stressors over 2 days induces chronic stress.

### Gene expression in muscle, fat body and CNS

To help determine how muscle responds to pathogen presence under the five different treatments, we examined the expression of a suite of genes in muscle, CNS and fat body (see Supplementary Materials and Methods). Samples were collected over several weeks, representing different generations of *M. sexta*. All tissues were handled in accordance with current guidelines for maintaining sample quality (Taylor et al., 2010). Tissue samples were stored in RNALater at −80°C until extraction.

RNA extraction and real-time quantitative PCR (qPCR) were performed as described previously (McMillan and Adamo, 2020) and summarized here. RNA extraction was performed using the RNeasy lipid tissue mini kit (Qiagen). All steps adhered to the manufacturer's instructions and included a DNase1 digest (RNase-Free DNaset, Qiagen) step to remove genomic DNA contamination. Concentration and integrity were determined using a Qubit 4.0 fluorometer (Invitrogen, Waltham, MA, USA). Purity was established using an Epoch microplate spectrophotometer (BioTek, Santa Clara, CA, USA). Only samples that adhered to the cutoffs outlined in the MIQE guidelines were used in analysis (Taylor et al., 2010). For more detailed methodology, please see the Supplementary Materials and Methods.

### qPCR and reference gene assessment

To determine the relative expression of the genes, cDNA levels were measured by qPCR. Details on primer sets and efficiencies can be found in Table S1. Standard curve analysis and temperature gradient analysis were conducted on each primer pair in each tissue to determine efficiencies and optimal temperature. Each primer set was tested for specificity within each tissue (see Supplementary Materials and Methods).

cDNA concentration was normalized in all samples prior to amplification. qPCR was conducted using SsoAdvanced Universal SYBR Green Supermix (Bio-Rad). qPCR reactions consisted of two technical replicates per sample, a positive control, a no-template control, and an interpolate calibrator for each plate and gene combination. Melt curves and melt peak analyses were done to confirm a single product at the end of each qPCR run.

Four potential reference genes were selected from previous studies on *M. sexta* (McMillan et al., 2018) and tested for stability across all groups: *ribosomal protein L17a* (*RpL17a*), *ribosomal protein S3* (*MsS3*), *ubiquitin* and *glycerol-3-phosphate dehydrogenase* (*G3PDH*). We found that the genes that gave the most stable baseline were *ubiquitin* and *RpL17a*. For more details, see Supplementary Materials and Methods.

### Muscle glycogen content

After receiving one of the five treatments – immune challenge, predator challenge, combined challenge, sham-injection or control – caterpillars were frozen at −80°C. Rapid freezing of the tissue reduced glycogen breakdown during dissection (Fournier et al., 2004). Caterpillars were dissected on ice to prevent thawing. The exoskeleton (cuticle) was peeled from the dorsal side and multiple fibres of intersegmental body wall muscles (Tsuji et al., 2020) were removed. Any fat body and other tissues were removed from the muscle.

Glycogen was measured using methods adapted from Tennessen et al. (2014). Briefly, the muscle tissue was homogenized and incubated in 100 µl of phosphate-buffered saline (PBS, P4417, Sigma-Aldrich, St Louis, MO, USA) at 70°C for 10 min. Incubation allowed for denaturation of any glycogen phosphorylase involved in glycogen breakdown (Meyer-Fernandes et al., 2000a,b). Each sample was then centrifuged at 14,000 rpm for 5 min at 4°C. Supernatant was stored at −80°C. Samples were thawed and divided into three aliquots. One aliquot was used to measure total protein using a Bradford assay. The second aliquot was used to measure the total free glucose in the muscle using a glucose assay kit (GAHK20, Sigma-Aldrich), following the manufacturer's instructions. In the third aliquot, the enzyme amyloglucosidase (Sigma-Aldrich; 2 mg ml^−1^) was used to convert all the glycogen into glucose. Samples, controls and glycogen standards were incubated with the enzyme at 37°C for 1 h and then assayed for glucose as described above. A pilot study determined that the 1 h incubation allowed the enzyme to fully convert all the muscle glycogen to glucose.

Absorption was measured at 340 nm wavelength. To determine the glycogen concentration in the muscle, the baseline glucose concentration was subtracted from the sample's total glucose value in the glycogen assay. Values were then normalized to mg protein, determined using the Bradford assay (see details in Adamo et al., 2016). The experiment was repeated twice, about 4 months apart.

We also tested whether production of anti-predator behaviour reduced muscle glycogen. Fifth instar, day 2 caterpillars were given a mock predator challenge as described above (*n*=7), then frozen and dissected immediately after the challenge. Control caterpillars (*n*=7) were frozen and dissected at the same time.

### Measurement of the maximal force of the defensive strike

To measure the force of a defensive strike, we developed an apparatus fitted with an accelerometer (Supplementary Materials and Methods) whose output was recorded and processed using Matlab (v. 9.6.0, MathWorks). A defensive strike was triggered by pressing a 4.74 N Von Frey filament perpendicularly against the body wall just above the left A6 leg. Pilot studies showed that a 4.74 N Von Frey filament reliably produced a defensive strike without inducing any damage to the exoskeleton.

Three hours after the delivery of the final treatment, the force of the defensive strike was measured for animals in each of the five treatments. Defensive strikes were induced and recorded 3 times and the median force was used in the analysis. The *Manduca* meter was cleaned with ethanol after each use. The person running the assay and recording the defensive strike was blind to the caterpillar's treatment group. The experiment was run over several months, using several cohorts (i.e. generations) of caterpillars.

### Test of a caterpillar's ability to repel attacking wasps

For the predator attack experiment, 25–50 *C. congregata* wasps were placed in a 30 cm^3^ mesh cage (Bugdorm^®^). A single fourth instar caterpillar was placed on a piece of green construction paper (approx. 10 cm×10 cm) and placed inside the mesh cage. Caterpillars were from one of three treatments (sham, immune challenge or control). The person performing the wasp study was blind to the treatment of the caterpillars to prevent any bias in caterpillar positioning in the cage. The wasp attack was filmed. To count as a ‘wasp attack’, the wasp must have landed on the caterpillar and adopted the ovipositing posture. Wasp attacks typically elicit violent defence responses in the caterpillar (e.g. defensive strikes and swinging the body from side to side). After an attack, the caterpillar was removed, and replaced in its container. Caterpillars were considered to have successfully repelled an attack if no wasp larvae emerged from the caterpillar and the caterpillar was able to pupate (*M. sexta* caterpillars that are successfully stung by *C. congregata* never pupate; Beckage and Gelman, 2002). If caterpillars died prior to wasp emergence, they were dissected to determine whether wasp larvae were present. The study was performed once over several weeks using different generations of wasps and caterpillars.

### Data analysis

Data were analysed using SPSS (v. 25, 26, 27 and 28, IBM) and Prism (v. 9.4, Graphpad). Outliers were removed in accordance with Hoaglin and Iglewicz (1987). Data that did not fit the assumptions for a parametric test were analysed using non-parametric tests. Muscle glycogen samples with low Bradford protein values (less than 30 mg) were removed from the analysis. We also removed tissue samples with high glucose levels (i.e. more than 10%; Steele, 1981) suggesting breakdown during dissection.

Treatment groups were compared with control samples. Controls were used as the baseline instead of the sham group because wounding is known to induce an immune response (Wigby et al., 2008). Wigby et al. (2008) argue against the use of an injection sham as the baseline for immune studies because it can mask some immune effects. In all of our studies, the sham group was not significantly different from the controls.

The qPCR data were analysed using CFX Manager v. 3.1 (Bio-Rad). Data were calculated as fold-change in expression of target genes in treated caterpillars against control caterpillars using the Relative Expression Software Tool (REST 2009, v. 1) program. Intermediate absolute concentration values for each group were calculated using primer efficiency data and group Cq values (Concentration=efficiency^average Cq (controls)−average Cq (samples)^). Normalized expression of each gene was then calculated as the ratio of the concentration of the gene of interest versus the mean concentration of our two reference genes. To test for statistically significant gene expression levels for each group relative to control (i.e. relative expression), we performed independent pairwise fixed reallocation randomization tests (the default statistical test implemented by REST 2009). Each of these tests was performed using 10,000 iterations. The 95% confidence intervals (CIs) for each group were determined via bootstrapping, based on the sorted expression ratios following random reallocation tests.

## RESULTS

### Gene expression during exposure to predators and pathogens

**Fig. 1.**
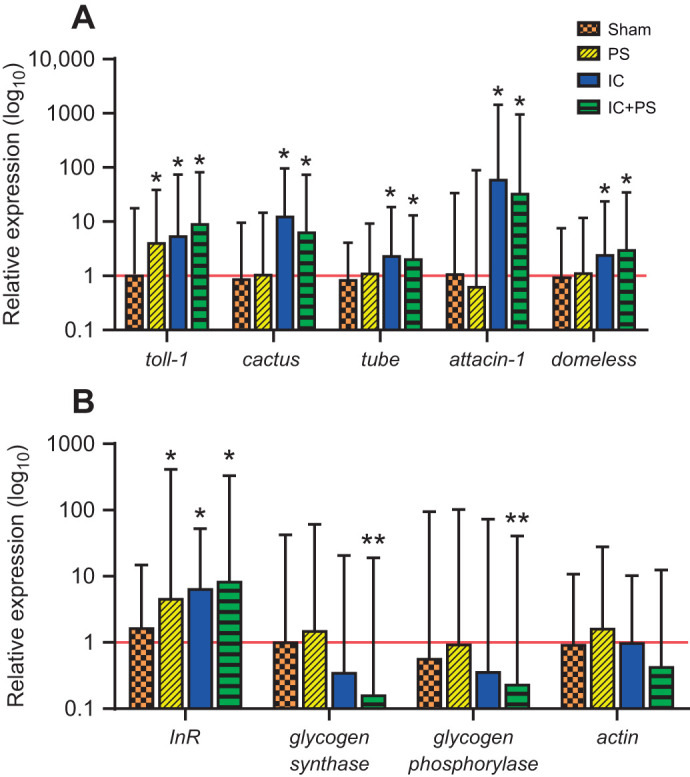
**Expression in muscle varies for immune- and metabolism-related genes in *Manduca sexta* caterpillars.** Bars represent mean immune- (A) and metabolism-related (B) gene expression relative to that in the control group (*n*=10) and error bars represent the upper 95% confidence interval (CI). Asterisks represent values significantly (*P*<0.05) upregulated (single asterisks) and downregulated (double asterisks) from those of controls, which have been normalized to 1 (red line). Sham: *toll-1* and *insulin receptor* (*InR*), *n*=9; *cactus*, *tube*, *attacin-1* and *domeless*, *n*=11; *glycogen synthase* and *glycogen phosphorylase*, *n*=10. Predator stress (PS): *cactus*, *tube*, *attacin-1*, *domeless* and *InR*, *n*=10; *toll-1*, *n*=9; *glycogen synthase* and *glycogen phosphorylase*, *n*=10. Immune challenge (IC): *toll-1*, *cactus*, *tube*, *attacin-1*, *domeless*, *InR*, *glycogen synthase* and *glycogen phosphorylase*, *n*=10. Combined immune challenge and predator stress (IC+PS): *toll-1*, *cactus*, *tube*, *attacin-1*, *domeless*, *InR*, *glycogen synthase* and *glycogen phosphorylase*, *n*=10. *Actin* assays: *n*=10 for all groups. Exact *P*-values are given in Table S2.

**Fig. 2.**
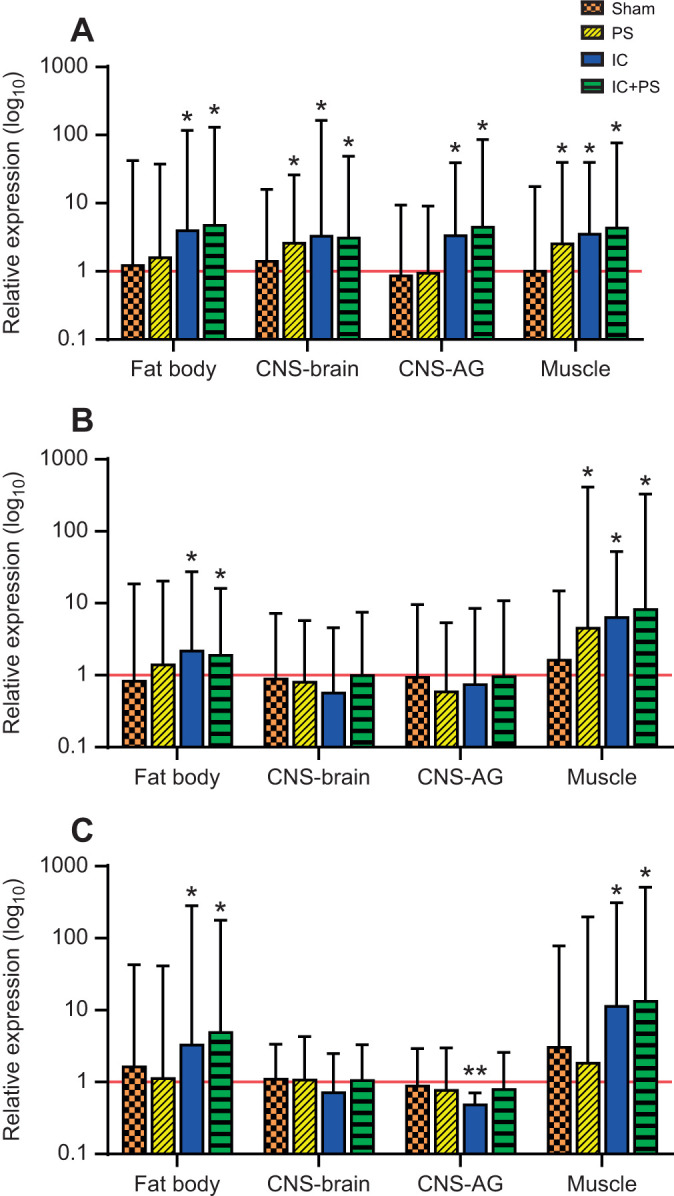
***toll-1*, *InR* and *GST-1* expression changes in the fat body, muscle and CNS of *M. sexta* caterpillars.** Bars represent mean *toll-1* (A), *InR* (B) and *GST-1* (C) gene expression relative to control (*n*=10) and error bars represent the upper 95% CI. Asterisks represent values significantly (*P*<0.05) upregulated (single asterisks) and downregulated (double asterisks) versus control (which was normalized to 1; red line). (A) *toll-1*: fat body: sham and PS, *n*=9; IC and IC+PS, *n*=10; CNS-brain: *n*=10 all groups; CNS-abdominal ganglia (AG): *n*=10 all groups; muscle: sham and PS, *n*=9; IC and IC+PS, *n*=10. (B) *InR*: fat body: sham, *n*=9; *n*=10 all other groups and tissues. (C) *GST-1*: fat body: *n*=9 all groups; all other tissues: *n*=10 all groups. Exact *P*-values are given in Table S2.

After an immune or combined challenge, muscle showed an increase in the expression of genes involved in the immune response, as well as in *GST-1* (Figs 1 and 2). There was no change in expression of *glycogen synthase* or *glycogen phosphorylase* genes with either predator or pathogen exposure, but a combined presentation resulted in the downregulation of expression of both genes (Fig. 1). In contrast, the *insulin receptor* (*InR*) gene showed increased expression in muscle with exposure to predators, pathogens and the combined challenge (Figs 1 and 2). Gene expression for the muscle-related protein actin was unchanged by any treatment. Sham treatment had no effect on the expression of any of the genes tested (Figs 1 and 2).

The fat body responded to an immune challenge or the combined challenge with increased *toll-1* receptor gene expression (Fig. 2A) as expected. An immune challenge also induced increased expression of both *InR* and *GST-1* (Fig. 2B,C).

Different parts of the CNS showed distinctive patterns of gene expression in response to predator stress or pathogen presence (Fig. 2). The brain showed increased expression of *toll-1* in response to predator and/or pathogen exposure (Fig. 2A). The abdominal ganglia showed no significant change in expression in response to predator stress, but did show an increase in *toll-1* expression in response to pathogen exposure or a combined challenge (Fig. 2A).

### Effects of immune challenge on muscle glycogen content

**Fig. 3.**
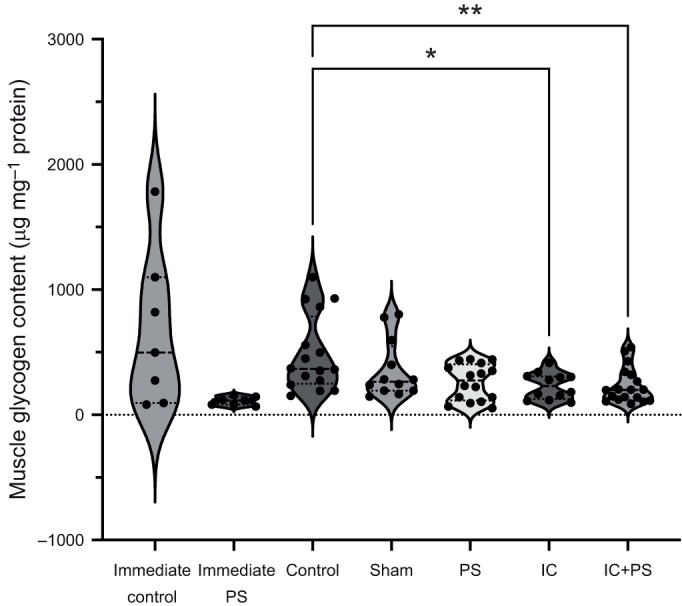
**Effects of immune and predator challenge on muscle glycogen content.** Muscle glycogen declined immediately after repeated defensive strikes (immediate PS; *n*=7) compared with controls (immediate control; *n*=7). Muscle glycogen also declined during immune challenge (IC, *n*=12) and during the combined challenge (IC+PS; *n*=18) relative to controls (*n*=16). Sham treatment (*n*=12) and predator stress (PS; *n*=16) alone had no effect. The dashed horizontal lines within each violin plot represent medians while the dotted lines denote the 1st and 3rd quartiles. The dots represent individual data points. Asterisks represent significance (*P<0.05, **P<0.01; see Results for statistical details).

Glycogen content in the muscle varied across the treatment groups (Fig. 3; Kruskal–Wallis=11.92, *P*=0.018). Both the immune challenge (Dunn's multiple comparison test, *Z*=2.62, adjusted *P*-value=0.036, *n*=12) and the combined challenge (Dunn's multiple comparison's test, *Z*=3.04, adjusted *P*-value=0.01, *n*=18) reduced glycogen in the muscle compared with controls (*n*=16), whereas sham injection (Dunn's multiple comparison's test, *Z*=1.17, adjusted *P*-value=0.96, *n*=12) and predator stress (Dunn's multiple comparison's test, *Z*=2.32, adjusted *P*-value=0.082, *n*=16) did not. However, glycogen content in muscle was reduced immediately after a mock predator challenge (Mann–Whitney=9.0, *P*=0.05, *n*=7 predator exposure, *n*=7 control).

### Force of the defensive strike

**Fig. 4.**
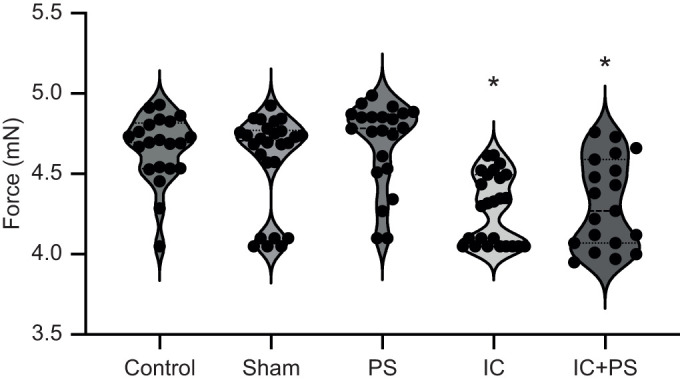
**Effects of immune and predator challenge on defensive strike force.** The force of the defensive strike declined during an immune challenge (IC; *n*=25) and during the combined challenge (IC+PS; *n*=19) relative to that of controls (*n*=21). Sham treatment (*n*=26) and predator stress (PS; *n*=22) alone had no effect. The dashed horizontal lines within each violin plot represent medians while the dotted lines denote the 1st and 3rd quartiles. The dots represent individual data points. The asterisk denotes a significant difference from controls (*P*<0.01, see Results for statistical details).

Immune challenge (*n*=25) and the combined predator stress plus immune challenge (*n*=19) resulted in a decline in the maximal force of the defensive strike compared with controls (*n*=21), even after controlling for caterpillar mass [Fig. 4; *F*_4,107_=14.9, *P*<0.001, mass used as a covariate in the analysis; pairwise comparisons against control (LSD), immune challenge<control, *P*<0.001, combined challenge<control, *P*<0.001]. The force of the defensive strike of caterpillars exposed to predator stress (*n*=22, *P*=0.70) or of the sham-treated controls (*n*=26, *P*=0.34) was not significantly different from that of controls. Although immune-challenged caterpillars experience illness-induced anorexia (Sullivan et al., 2016), their mass was not significantly different from that of controls (Fig. S1; *F*_4,107_=2.12, *P*=0.08). We examined control caterpillars to determine whether there was a correlation between mass and defensive strike force. We found a positive correlation (Pearson's correlation, *r*^2^=0.44, *P*=0.014, *n*=21).

### Test of a caterpillar's ability to repel attacking wasps

Activating the immune system also led to a reduction in the ability of fourth instar caterpillars to repel wasps. Using a planned comparisons analysis for rankable data (Meddis, 1984), immune-challenged caterpillars were significantly more likely to be successfully attacked by wasps (53.2%, 25/47), than were control (40.3%, 27/67) and sham-treated (42%, 21/50) caterpillars (*Z*=2.83, *P*=0.002; Fig. S2).

## DISCUSSION

Muscle reacted to an immune challenge with an increase in the expression of genes involved in the immune response (Fig. 1), even though muscle is not usually thought of as an immune organ. Genes for proteins involved in the toll signalling pathway (*toll-1*, *tube* and *cactus*) and Jak/Stat pathway (*domeless*) were upregulated in muscle during exposure to heat-killed pathogens (Fig. 1). Although many molecules have multiple functions (e.g. Toll receptors; Umetsu, 2022), it appears that muscle did mount an immune-like response, resulting in increased transcription of the gene *attacin-1*, which encodes an antimicrobial peptide induced by activation of the toll signalling pathway in *M. sexta* (An et al., 2010). This result suggests that muscle in *M. sexta* may be capable of producing and secreting AMPs (Chatterjee et al., 2016) and antimicrobial proteins (Eleftherianos et al., 2021b), as has been found in *Drosophila*. Moreover, the induction of these immune genes was specific to pathogen exposure. Except for the gene for the toll-1 receptor, immune genes (e.g. *attacin*) were not upregulated by predator stress.

The participation of muscle in the immune response appeared to come at a cost. The caterpillars' main anti-predator behaviour, the defensive strike, showed decreased force (Fig. 4). Caterpillars were less able to defend themselves against one of their common enemies, the wasp *C. congregata*, suggesting that their decline in survival is due to the decline in anti-predator behaviour. Although we do not know why there was a decline in defensive strike behaviour, we have evidence that muscle lost resources during an immune challenge. The amount of glycogen, a key energy resource, declined (Fig. 3). Glycogen content also declined immediately after a mock predator attack, suggesting that anti-predator behaviour is resource demanding as well. This parallels results in mammals, in which exercise reduces muscle glycogen content (Jensen et al., 2011). Therefore, our results show that intersegmental muscle in *M. sexta* responds to an immune challenge. Muscle may: (1) assist with immune defence by the production of AMPs and/or, (2) promote a more robust immune response by increasing resource availability to the immune system. Muscle suffers a decline in function during this response.

The use of muscle glycogen for both anti-predator behaviour and an immune response raises the possibility of physiological trade-offs between the two functions. Such a trade-off could help explain the increase in disease susceptibility in insects after energetically demanding behaviours such as flight (Adamo and Parsons, 2006; Adamo, 2012). Physically demanding behaviours reduce the amount of glycogen in muscle, and less glycogen in muscle leads to greater susceptibility to infection in *Drosophila* (Yang and Hultmark, 2017). Similarly, the reduction in muscle glycogen during an immune challenge may play a role in the reduction in anti-predator behaviour. The existence of these trade-offs suggests that marshalling resources from across the body is critical when an animal is facing life-threatening events. Given the costs, muscle's participation in immunity must provide critical benefits (e.g. Yang and Hultmark, 2017); it would be unlikely to occur across species if it ultimately reduced fitness.

The details of how an immune challenge results in reduced glycogen in muscle are not known. We found no evidence of an upregulation of glycogen phosphorylase gene expression during an immune challenge, and therefore we have no evidence for increased glycogen breakdown. However, glycogen in muscle might decline if glucose consumption by muscle is reduced. In *Drosophila*, cytokines can induce insulin resistance (Dolezal et al., 2019), and insulin resistance could occur despite the increase in *InR* transcription in muscle. A reduction in muscle's consumption of glucose may be critical for a robust immune response in our food-restricted caterpillars. Food-restricted caterpillars have less glucose in their haemolymph (Adamo et al., 2016), and glucose is required for effective cell-mediated immunity (Bajgar et al., 2015). More studies are needed on how nutrition shapes inter-organ connections during an immune response.

Immune challenge could reduce muscle function in a variety of ways, in addition to reducing glycogen stores. In larval *Drosophila*, exposing body wall muscle to lipopolysaccharides leads to muscle hyperpolarization and a reduction in the amplitude of the excitatory junction potential (Istas et al., 2019). These changes appear to be mediated via receptors on muscle for immune signalling molecules (Istas et al., 2019). Similar events could contribute to the decline in the force of the defensive strike observed in our study, as gene expression for receptors for immune signalling molecules (e.g. *toll-1* and *domeless*) occurs in *M. sexta* muscle. Immune-generated damage of muscle can also depress its function. When exposed to bacteria, larvae of the damselfly, *C. puella*, show evidence of oxidative damage to the abdominal muscles used for swimming, with concomitant slower escape swimming speeds (Janssens and Stoks, 2014a,b). We have no direct evidence that this is occurring in *M. sexta*; however, we did find an upregulation of expression for *GST-1* in muscle during an immune challenge or a combined challenge (Fig. 2). The enzyme GST can protect tissues against oxidative damage (Liu et al., 2016), and might be expected to be upregulated, if muscle were being damaged during an immune challenge.

There is no evidence that the decline in the force of the defensive strike is due to a reduction in the motivation for defensive behaviour. In fact, an immune challenge decreases the stimulus threshold needed to activate a defensive strike in *M. sexta* (Adamo and McMillan, 2019), suggesting that caterpillars are not less motivated to perform anti-predator behaviour during infection. This increase in nociceptive sensitivity may help compensate for the diminished force of the defensive strike. Anti-predator behaviour may also decline during an immune challenge as a result of the indirect effects of infection as opposed to direct effects on muscle per se. Immune challenge results in illness-induced anorexia in most insects (Sullivan et al., 2016), including *M. sexta* (Adamo, 2005). Although we did not find that immune-challenged caterpillars weighed less than controls in our laboratory-reared caterpillars (Fig. S1), in the field, attacks from live pathogens are likely to cause caterpillars to lose weight (Sullivan et al., 2016). In our study, mass was positively correlated with the force of the defensive strike, suggesting that illness-induced anorexia could carry a cost in terms of increased predation risk by decreasing caterpillar mass gain. This cost would be in addition to any direct effects on muscle.

The results in this paper extend recent work showing that there is a whole-organism response to an immune challenge (e.g. Dolezal et al., 2019; Galenza and Foley, 2019; Zhang et al., 2021; Adamo, 2022; Yu et al., 2022). An immune challenge induces physiological (Dolezal et al., 2019; Yu et al., 2022) and behavioural (Sullivan et al., 2016) changes, optimizing the body for immune defence. This immunophysiological response shows parallels with the flight-or-fight response that occurs with exposure to predators (Dolezal et al., 2019; Adamo, 2022). Predator stress also induces long-lasting physiological and behavioural changes in *M. sexta* (Thaler et al., 2012; McMackin et al., 2016; Adamo et al., 2017; Tabuena et al., 2017) and other insects (Cinel et al., 2020), as well as in vertebrates (e.g. Harris and Carr, 2016). Both responses function as part of an integrated defence system (Adamo, 2020, 2022). The existence of connections across different defence systems helps explain the upregulation of *toll-1* gene expression during predator stress in both muscle and brain (Fig. 2A). Specific stressors (e.g. predator stress) typically induce the upregulation of multiple stress responses (Adamo, 2022). These upregulated components of different defence systems may be important in maintaining different types of defence when resources are being siphoned away to fuel the response to a current challenge (Adamo, 2022). Muscle plays multiple roles in this integration across defence systems. It participates in both anti-predator and immune responses, and it also helps regulate energy metabolism across the organism depending on the physiological context (Alfa and Kim, 2016; Bretscher and O'Connor, 2020; Zhao and Karpac, 2021; Yu et al., 2022).

When co-activated, both the fight-or-flight response and immune response are altered compared with when activated alone (Adamo, 2020, 2022; Table 1). From the perspective of the immune system, a combined challenge produces a complex mix of changes to the response to pathogens, with some immune components increasing in function (e.g. constitutive gene expression for *attacin-1*; Adamo et al., 2017), some decreasing (e.g. phenoloxidase; Janssens and Stoks, 2014b; see also Adamo, 2020) and some remaining unchanged (Adamo et al., 2017) compared with the immune response without predator stress (Adamo, 2020). Therefore, the immune response is context dependent, and alters its configuration in the presence of predators (Adamo, 2020). We expected that muscle's response would also be context dependent, and that it would decrease its contribution to the immune response during periods of chronic predator stress. However, we found that chronic predator stress seemed to have little impact on muscle's contribution to the immune response in terms of immune gene expression (Table 1), defensive strike force (Fig. 4) or glycogen muscle content (Fig. 3). The only significant difference we did find between the single challenges and a combined challenge was that a combined challenge resulted in the downregulation of expression of both *glycogen synthase* and *glycogen phosphorylase* in muscle (Fig. 1). The downregulation of both genes may help ensure that muscle resources are used to fuel immediate needs (i.e. not used to make the storage molecule glycogen) and, at the same time, help muscle maintain some resources (e.g. for anti-predator behaviour). We might have found more evidence for our prediction if we had tested the acute, rather than the chronic, effects of predator stress on muscle's response to an immune challenge.

**Table 1.**
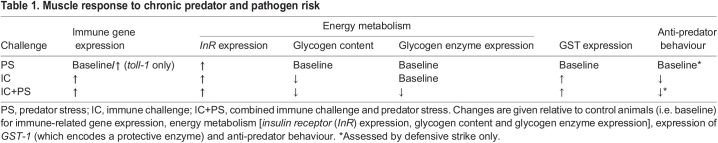
Muscle response to chronic predator and pathogen risk

It is impossible to infer the details of an organ's response from the expression of three genes. Nevertheless, our results do show that both the fat body and CNS respond to predators, pathogens and combined challenges in different ways (Fig. 2). In the fat body, one of the insect's main immune organs, exposure to pathogens increased expression of *toll-1*, as has been observed previously in *M. sexta* (Gunaratna and Jiang, 2013; Cao et al., 2015). In the brain, *toll-1* gene expression increases in response to both predators and pathogens (Fig. 2). In *Drosophila*, Toll-1 is involved in the response to both DAMPs and PAMPs within the CNS (Shmueli et al., 2018). Possibly the brain of *M. sexta* increases its sensitivity to these signals during periods of predator and pathogen exposure, although establishing this would require further study.

The decline in anti-predator behaviour during an immune response is a non-immunological cost of infection, similar to non-consumptive effects of predators (e.g. Hermann and Landis, 2017). Exposure to predators produces a range of negative impacts in insects (Hermann and Landis, 2017), including *M. sexta* (Thaler et al., 2012). However, non-immunological costs of infection are underappreciated (Nadler et al., 2023), but can have a large impact on animal fitness (Lopes, 2023). We show that increased mortality from predation is a likely non-immunological cost of infection in *M. sexta*. Our study also suggests that one reason non-immunological costs exists is the participation of diverse organs, such as muscle, in immunity.

## Supplementary Material

10.1242/jexbio.245861_sup1 Supplementary information
