# Supplementary material for: Muscle in the caterpillar Manduca sexta responds to an immune challenge, but at a cost, suggesting a physiological trade-off
Source: J Exp Biol. 2023 Jul 25;226(14):jeb245861. doi: 10.1242/jeb.245861 (PMC10399994; doi:10.1242/jeb.245861)
Supplement: Supplementary information [file jexbio-226-245861-s1.pdf]

Supplementary Materials and Methods

Real-Time Quantitative Polymerase Chain Reaction and Reference Gene Assessment:

All samples were handled in accordance with current guidelines for maintaining sample quality (Taylor et al., 2010; Bustin et al., 2009). Prior to dissection, larvae were placed in a -20 °C freezer for 10 minutes. Cuticle was peeled from the dorsal side of the abdomen, and intersegmental muscle, CNS and fat body was removed. Samples were stored in RNALater at -80° C until extraction.

RNA extraction and qPCR were performed as described previously (McMillan and Adamo, 2020) and is repeated here. RNA extraction was performed using the RNeasy lipid tissue mini kit (Qiagen). All steps adhered to the manufacturer's instructions and included a DNase1 digest (RNase-Free DNaset, Qiagen) step to remove genomic DNA contamination. 5 uL of the resulting RNA was immediately used for generation of complementary DNA (cDNA). The concentration and purity of the extracted RNA was determined using an Epoch microplate spectrophotometer (BioTek, Santa Clara, Ca, USA). Only samples with an A260/A280 ratio greater than 2.0 were used, indicating high purity RNA (Taylor et al., 2010). The integrity of the extracted RNA was determined using a Qubit 4.0 fluorometer and the Qubit RNA IQ Assay Kit (Invitrogen, Waltham, MA, USA). Only samples with a score of 8.5 or higher were used, indicating a high degree of integrity and quality according to the manufacturer's guidelines. The remaining volume was stored at -80° C.

To generate cDNA, 5 uL of the RNA was mixed with 11 uL of RNase-free water and 4ul of 5x iScript Reaction Mix (Bio-Rad) in a chilled PCR tube (Bio-Rad). Samples were heated at 25 ° C for 5 min, 42 ° C for 30 min and 85 ° C for 5 min using a c1000 Thermal Cycler (Bio-Rad). cDNA concentration was measured using a Qubit 4.0 fluorometer and Qubit dsDNA HS Assay kit (Invitrogen, Waltham, MA, USA). The resulting cDNA was stored at -80° C.

To determine the relative expression of the genes, cDNA levels were measured by quantitative real-time polymerase reaction (qPCR). Details on primer sets and efficiencies can be found in Table S1. Standard curve analysis, and temperature gradient analysis were conducted on each primer pair in each tissue to determine efficiencies and optimal temperature. Each primer set was tested for specificity in each tissue by running products out on an agarose gel, cutting out the bands and extracting the product using a QIAquick Gel Extraction Kit (Qiagen). The

Journal of Experimental Biology • Supplementary information

resulting products were sent away to be sequenced using Sanger sequencing (GeneWiz, Burlington, MA, USA). The resulting sequences were run through BLAST to check identity. Primers were purchased from Integrated DNA Technologies (http://www.idtdna.com/site).

cDNA concentration was normalized in all samples prior to amplification. For each sample and target gene combination, 4 µL of sample was mixed with 5 µL SsoAdvanced Universal SYBR Green Supermix (Bio-Rad), 0.4 µL of distilled water, and 0.3 µL of 10 µM forward and reverse primer, for a 10 µL reaction mixture. A c1000 Thermal Cycler and CFX96 Real-Time system (Bio-Rad) were used for cycling, set for 1 cycle at 95° C for 3 min, and 45 cycles of denaturation at 95 for 15 s, annealing at 60° C for 30s and extension at 72° C for 30 s. CFX Manager (Bio-Rad) was used to calculate quantitative cycle (Cq) values, with a cut off at 35 Cq. qPCR reactions were performed using two technical replicates per sample, a positive control, a no template control, and an interpolate calibrator for each plate and gene combination. A melt curves and melt peak analyses were done to confirm a single product at the end of each qPCR run.

Four potential reference genes were selected from previous studies on *M.sexta* (McMillan et al., 2018) and tested for stability across all groups: ribosomal protein L17a (RpL17a), ribosomal protein S3 (MsS3), ubiquitin, glycerol-3-phosphate dehydrogenase (G3PDH). Three samples from each treatment and tissue combination were used for this assessment. qPCR temperature parameters, reaction mixtures and primer regulations followed the protocol used for target genes.

Table S1. Forward and reverse primer sequences, efficiencies, and reference for target genes

**Gene**	**Tissue**	**E %**	**Reference**
Actin<br>F:5’CTCTTCCAGCCTTCCTTCCT-3’<br>R:5’ACAGGTCCTTACGGATGTCG-3’	Fat body	99	Schwartz et al., 1993
	Muscle	100	
	Supraesophageal	101	
	Abdominal	101	
Attacin-1<br>F:5’GCAGGCGACGACAACAAC-3’	Fat body	97	An et al., 2010
	Muscle	99	

Journal of Experimental Biology • Supplementary information

R:5’ATGCGTGTTGGTAAGAGTAGC-3’	Supraesophageal	98	
	Abdominal	98	
Cactus<br>F:5’ TTCGGGTGAAGTGGATTCGG-3’<br>R:5’ TCGTTGTCAGGGTGGAACAG-3’	Fat body	92	Based on CDS from Cao et al., 2015
	Muscle	93	
	Supraesophageal	95	
	Abdominal	95	
Domeless<br>F: 5’GATGTGTCCAGTTTGCCAGC-3’<br>R:5’ TTTGGCCTTTCACTGGTGGT-3’	Fat body	98	Based on CDS from Cao et al., 2015
	Muscle	100	
	Supraesophageal	99	
	Abdominal	99	
InR<br>F:5’GACTTAGCGGCACGTAATTGCATGG-3’<br>R: 5’TACCCCGTCTTTCAAACTCTCG-3’	Fat body	105	Cao et al., 2015
	Muscle	102	
	Supraesophageal	105	
	Abdominal	105	
Glycogen Synthase<br>F:5’ AGGTCCAAACTAACGGCGAG-3’<br>R:5’ CGTTCGGCGTTCGGTAATTC-3’	Fat body	102	Based on NCBI reference sequence: XM_030173055.2
	Muscle	102	
	Supraesophageal	103	
	Abdominal	103	
Glycogen Phosphatase<br>F:5’ ACCTGACGATTGGCTTCGTT-3’<br>R:5’ ATCGTAGGGCATGGCAAACA-3’	Fat body	99	Based on NCBI reference sequence: XM_30176289.2
	Muscle	99	
	Supraesophageal	98	
	Abdominal	98	
GST-1<br>F:5’AAGTACCCGTTCCAGCTGAA-3’<br>R:5’ TGGGTTGGACAGGACAGTTT-3’	Fat body	100	McMillan et al., 2018
	Muscle	99	
	Supraesophageal	97	
	Abdominal	97	
RpL17a<br>F:5’ TCCGCATCTCACTGGGTCT-3’<br>R:5’ CACGGCAATCACATACAGGTT-3’	Fat body	101	Rewitz et al., 2006
	Muscle	100	
	Supraesophageal	100	

Journal of Experimental Biology • Supplementary information

	Abdominal	100	
Toll-1<br>F:5' CGCAACAATACCTACGTGCG-3'<br>R:5' GGGTACACGCAAGTTCTGGA-3'	Fat body	105	Based on NCBI reference sequence: XM_037441618.1
	Muscle	103	
	Supraesophageal	103	
	Abdominal	103	
Tube<br>F:5' GGAAATCGACAATGGTTGGCA-3'<br>R:5' AACCTCTGCACACTTTCGGT-3'	Fat body	101	Based on CDS from Cao et al., 2015
	Muscle	100	
	Supraesophageal	100	
	Abdominal	100	
Ubiquitin<br>F:5' AAAGCCAAGATTCAAGATAAG-3'<br>R:5' TTGTAGTCGGATAGCGTGCG-3'	Fat body	102	Kumar et al., 2012
	Muscle	103	
	Supraesophageal	101	
	Abdominal	101	

Table S2. Exact p-values for Gene Expression Values

**MUSCLE**	Dual	Immune	Predator	Sham
Glycogen	0.03	0.3	0.97	0.53
phosphatase	0.02	0.182	0.477	0.925
Glycogen synthase	0.01	0.01	0.63	0.98
Domeless	0.03	0.03	0.64	0.7
Tube Cactus	0.0001	0.0001	0.747	0.835
Attacin	0.0001	0.0001	0.6	0.82
Actin	0.15	0.94	0.26	0.95

**GST-1**	Dual	Immune	Predator	Sham
Fatbody	0.02	0.04	0.78	0.44
Muscle	0.002	0.002	0.71	0.34
Brain	0.26	0.09	0.43	0.18
A.G.	0.17	0.003	0.17	0.68

**InR**	Dual	Immune	Predator	Sham

Journal of Experimental Biology • Supplementary information

Fatbody	0.04	0.03	0.38	0.79
Muscle	0.0001	0.001	0.008	0.28
Brain	0.84	0.09	0.59	0.86
A.G.	0.96	0.53	0.17	0.99

**Toll-1**	Dual	Immune	Predator	Sham
Fatbody	0.006	0.02	0.33	0.6
Muscle	0.007	0.002	0.0001	0.36
Brain	0.03	0.03	0.01	0.33
A.G.	0.02	0.02	0.99	0.86

A.G. – abdominal ganglion

Glycogen Muscle Assay

Most of the caterpillars in the muscle glycogen study were fed on Frontier food as described. However, some caterpillars were fed on *M. sexta* diet made by Great Lakes Hornworm with the addition of 9 g of nutrient agar to 250 mL of diet (Oxoid, Nepean, ON) during the first 4 instars. As with the Frontier diet, the Great Lakes Hornworm diet was diluted 1:3 with non-nutritive cellulose once the caterpillars molted to the 5th instar. The number of caterpillars fed Great Lakes Hornworm were: Control – 3/16, Sham – 2/12, Immune Challenged – 1/12, Predator Stress – 2/16, Combined Challenge – 3/18. The values for the caterpillars fed the Great Lake Hornworm diet were all within the 1st and 3rd quartile for each treatment group.

Journal of Experimental Biology • Supplementary information

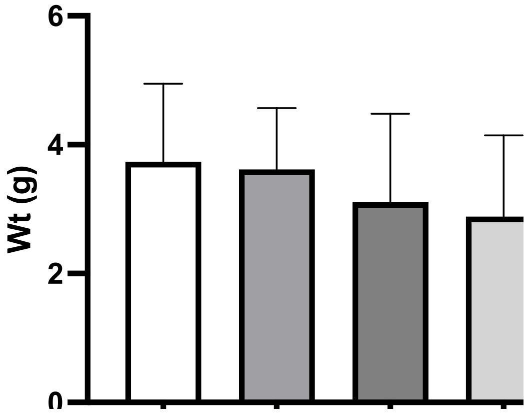

Fig. S1. Weights of caterpillars on the 5th Day 2, the day of the defensive strike test. There were no significant differences across groups (F(4, 107)=2.12, p=0.08. The bars represent means and the error bars denote one standard deviation. Control, n=21; Sham, n=26; Predator stress (PS), n=22; Immune-challenged (IC); n=25; Combined challenge (PS+IC); n=19.

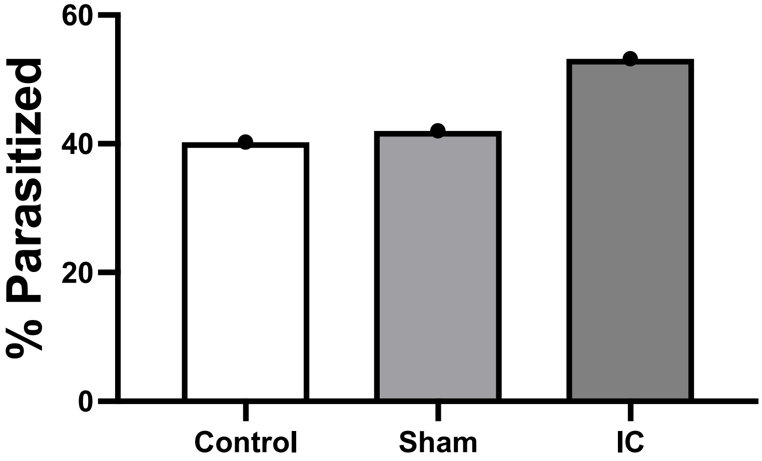

Fig. S2. Immune-challenged caterpillars are more likely to be parasitized by *Cotesia congregata* than controls (z=2.83, p=0.002). Bars represent the percent parasitized. Control n=67; Sham n=50; IC (Immune-Challenged) n=47.

Manduca Meter

The output of the Manduca meter was calibrated using von Frey hairs. Schematic for the Manduca meter and MatLab Code are available upon request.

Journal of Experimental Biology • Supplementary information
